# Delta opioid receptors on nociceptive sensory neurons mediate peripheral endogenous analgesia in colitis

**DOI:** 10.1186/s12974-021-02352-3

**Published:** 2022-01-06

**Authors:** Xavier Mas-Orea, Lilian Basso, Catherine Blanpied, Claire Gaveriaux-Ruff, Nicolas Cenac, Gilles Dietrich

**Affiliations:** 1 Digestive Health Research Institute (IRSD), Université de Toulouse, INSERM, INRA, ENVT, UPS, CHU Purpan BP 3028, 31024 Toulouse Cedex 3, France; 2 INFINITy, Université de Toulouse, INSERM, CNRS, UPS, Toulouse, France; 3 IGBMC, Université de Strasbourg, INSERM, CNRS, Illkirch, France

**Keywords:** Visceral pain, Opioid receptors, Nociceptors, T lymphocytes, Opioids, Intestinal inflammation

## Abstract

**Background:**

Inflammatory visceral pain is endogenously controlled by enkephalins locally released by mucosal CD4^+^ T lymphocytes in mice. The present study aimed at identifying opioid receptor(s) expressed on nociceptive sensory nerves involved in this peripheral opioid-mediated analgesia.

**Methods:**

The peripheral analgesia associated with the accumulation of CD4^+^ T lymphocytes within the inflamed colonic mucosa was assessed in conditional knockout mice specifically deleted for either of the two opioid receptors for enkephalins (i.e., µ (MOR) and δ (DOR) receptors) in Na_v_1.8-expressing sensory neurons in the dextran sulfate sodium (DSS)-induced colitis model.

**Results:**

Endogenous analgesia is lost in conditional knockout mice for DOR, but not MOR at the later phase of the DSS-induced colitis. The absence of either of the opioid receptors on sensory nerves had no impact on both the colitis severity and the rate of T lymphocytes infiltrating the inflamed colonic mucosa.

**Conclusion:**

The key role of DOR on primary afferents in relieving intestinal inflammatory pain opens new therapeutic opportunities for peripherally restricted DOR analgesics to avoid most of the side effects associated with MOR-targeting drugs used in intestinal disorders.

## Background

Management of non-cancer chronic pain with opioids has not any long-term benefits as dose escalation, associated with opioid tolerance, often results in addiction and overdose death [39, 47]. It has been estimated that 21% of outpatients with inflammatory bowel disease (IBD) are opioid users [30] and that 5% turn into heavy users [43]. In addition, opioid therapy induces intestinal adverse effects such as constipation and ileus that may lead to narcotic bowel syndrome, which paradoxically, makes pain worse, thereby leading patients into a vicious cycle of addiction. To limit opioid use disorders, a variety of strategies have been proposed including biased ligands [28, 38] or ligands specific for receptor splice-variant [27]. Because these approaches only partially overcome centrally mediated side effects such as addiction, alternative strategies based on the endogenous mechanisms of pain regulation in periphery are under investigation [8, 14, 26, 41]. They consist in locally sustaining the endogenous opioid activity [13, 35] or in activating peripheral opioid receptors [15, 25] that may be targeted only in an acidic inflammatory environment [2, 20, 23, 37, 40]. In this context, the finding that endogenous opioids locally produced by mucosal CD4^+^ T lymphocytes [4, 9–11, 44–46] alleviate inflammation-induced visceral pain represents an opportunity to improve opioid therapy. Beyond the potential therapeutic use of effector memory T lymphocytes including active vaccination [1, 5] or passive T cell transfer (immunotherapy) [24, 49], a better knowledge of the opioid receptor(s) involved in the peripheral analgesic effects of T lymphocytes might also refine prescription of opioid medications.

In this study, we compared the visceral sensitivity of mice with opioid receptor-deficient nociceptors to that of control opioid receptor floxed mice in the dextran sulfate sodium (DSS)-induced colitis model in which T lymphocyte accumulation induces analgesia [5, 9, 10, 23, 46].

## Methods

### Animals and experimental colitis

The MOR-floxed (*Oprm1*^fl/fl^) and DOR-floxed (*Oprd1*^fl/fl^) mouse lines were crossed with Na_v_1.8-Cre mice to produce conditional knockout (cKO) mice for MOR and DOR in afferent nociceptive neurons, respectively [16, 48]. All mice were on a mixed genetic background (C57BL6/J-SV129Pas) and were bred in the animal care facility at Toulouse (INSERM US 006 ANEXPLO/CREFRE, Toulouse, France).

Polymerase chain reaction (PCR) genomic DNA analyses were performed on DNA from dorsal root ganglia (DRG) neurons and brain to confirm *Oprm1* exon 2 and 3 deletion and *Oprd1* exon 2 deletion in DRGs and not the brain in cKO MOR and cKO DOR mice, respectively, as previously described [16, 48]. The forward primers A (5’-ACCAGTACATGGACTGGATGTGCC-3’) and C (5’-GTTACTGGAGAATCCAGGCCAAGCC-3’) and the reverse primers B (5’-TGCTAGAACCTGCGGAGCCACA-3’) and D (5’-CGCTTGGGAATATCTTGTACCTATGACCA-3’) were used to reveal *Oprm1* gene excision and intact floxed allele, respectively [48]. *Oprd1* gene excision and intact floxed allele were detected by using the forward primer E (5’-GGTTAGCCTTCTGAG GGCTGGG-3’) and the reverse primer F (5’-CCTGGCCAGCCAGTTCACAATCT-3’).

Mice used in the study were 8- to 12-week-old males weighing 20–25 g. They were housed by four in ventilated cages at a temperature between 20 and 22 °C and maintained under a 12 h light/dark cycle in sawdust-coated transparent cages with chow and water ad libitum. Controls were MOR or DOR-floxed littermate mice that did not express the Cre recombinase.

Colitis was induced by adding 3% (weight/volume) dextran sulfate sodium (DSS, 36,000–50,000 MW; MP Biomedicals, Illkirch, France) into the drinking water for 5 days. Then, from day 5 until day 12, animals received only water as previously described [10]. All procedures involving animals were performed in accordance with the Guide for the Care and Use of Laboratory Animals of the European Council and were approved by the Animal Care and Ethics Committee of US006/CREFE (CEEA-122; application number APAFIS #16385–2018080222083660 v3).

### Real-time quantitative RT-PCR analysis

Brain and DRG samples were homogenized in 500 µL TRIzol™ Reagent (Sigma). Total RNA was then isolated by using GenElute™ Mammalian total RNA miniprep Kit following the manufacturer’s instructions (Sigma). RNA was evaluated using a ND-1000 Nanodrop spectrophotometer and gel electrophoresis. RNA was then reverse-transcribed with Moloney murine leukemia virus reverse transcriptase using random hexamers for priming. Transcripts encoding hypoxanthine phosphoribosyl transferase (HPRT), Na_v_1.8, MOR and DOR were quantified by real-time quantitative polymerase chain reaction using the following forward and reverse primers: 5’-CATTGCTGACAGGATGCAGAAGG-3’ and 5’- TGCTGGAAGGTGGACAGTGAGG-3’ for *β-actin*, 5'- ATCGAAGCCAAGGAGAAGAAGTT-3' and 5'-CTGCCAGCACCTCCTGTT-3' for *SCN10A* (Na_v_1.8), 5’- GAGCCACAGCCTGTGCCCT-3’ and 5’-CGTGCTAGTGGCTAAGGCATC-3’ for *Oprm1*, 5’-GCTCGTCATGTTTGGCATC-3’ and 5’-AAGTACTTGGCGCTCTGGAA-3’ for *Oprd1*. The target gene expression was normalized to that of the β-actin and the 2^−ΔΔ*C*T^ method was used to evaluate mRNA expression levels relative to the corresponding littermate floxed control animals.

### Macroscopic assessment of colon injury

Macroscopic damage was evaluated using a scale ranging from 0 to 11: erythema (absent (0), length of the area less than 1 cm (1), more than 1 cm (2)), edema (absent (0), mild (1), severe (2)), strictures (absent (0), one (1), two (2), more than two (3)), ulceration (absent (0), present (1)), mucus (present (0), absent (1)), and adhesion (absent (0), moderate (1), severe (2)). Bowel wall thickness was measured with an electronic calliper at 0.5 cm above the anus (colorectum).

### Histological assessment of colon injury

Colonic tissue specimens were excised 0.5 cm proximal to the anus. Damage scoring was evaluated on 5-µm colonic sections stained with haematoxylin–eosin using a scale ranging from 0 to 12. Inflammatory cell infiltration, epithelial/mucosal alteration (including vasculitis, goblet cell depletion and crypt abscesses), mucosal architecture alteration (including ulceration and crypt loss) and submucosal edema were graded from 0 to 3 (absent, mild, moderate and severe).

### Assessment of mucosal lymphocyte density

Five-micrometer colonic sections deparaffinized, rehydrated and saturated with PBS 1% BSA were incubated with rabbit anti-CD3 (Clone SP7, Diagnostic BioSystems, Pleasanton, CA) monoclonal antibody for 1 h at room temperature. After washing with PBS, bound antibodies were revealed by further incubation with Alexa Fluor 555-labeled Donkey anti-rabbit IgG antibodies (Life Technologies, Carlsbad, CA). Slides were mounted and nuclei were stained with 4',6-diamidino-2-phenylindole (DAPI) fluorescent mounting medium (ProLong™ Gold, Invitrogen). Fluorescence images were taken using ApoTome Zeiss Axio-observer (Carl Zeiss Microscopy GmbH, Iéna, Allemagne) with × 20 objective. Colonic density of T lymphocytes was determined by counting anti-CD3 fluorescence cells relative to tissue surface delimited by using DAPI staining thanks to an algorithm running on Fiji software [10].

### Visceral sensitivity assessment by colorectal distension

Visceral sensitivity was examined by measuring the visceromotor response to colorectal distension. Three days before colorectal distension, 2 electrodes (Bioflex insulated wire AS631; Cooner Wire, Chatsworth, CA) were implanted into the abdominal external oblique musculature of mice previously anaesthetized with ketamine (10 mg kg^−1^) and xylazine (1 mg kg^−1^). Electrodes were exteriorized at the back of the neck and protected by a plastic tube attached to the skin. After surgery, mice were examined twice a day for abnormal behavior (absence of grooming, immobility) until the day of the distension. On the day of the distension, electrodes were connected to a Bio Amp, itself connected to an electromyogram acquisition system (ADInstruments, Inc., Colorado Springs, CO). Ten-second distensions were performed on conscious animals with a 10.5-mm-diameter balloon catheter (Fogarty catheter for arterial embolectomy, 4F; Edwards Lifesciences, Nijmegen, Netherlands) gently inserted into the colorectum at 5 mm from the anus (balloon covering a distance of 1 cm starting at 5 mm from the anus) and progressively inflated at pressures of 15, 30, 45 and 60 mm Hg with 5-min rest intervals. Electromyography activity of abdominal muscles was recorded to calculate visceromotor responses using LabChart 8 software (ADinstruments) [34].

### Statistical analysis

Data are expressed as mean ± SEM. The comparison between two groups of animals was performed using Mann–Whitney U test. Kruskal–Wallis test was used to compare three groups of mice. Comparison of visceral sensitivity in response to increasing distension pressures between animals were estimated with two-way repeated measurements analysis of variance (ANOVA). All statistical analyses were performed by using Prism 9.0 statistical software (GraphPad, San Diego CA).

## Results

**Fig. 1:**
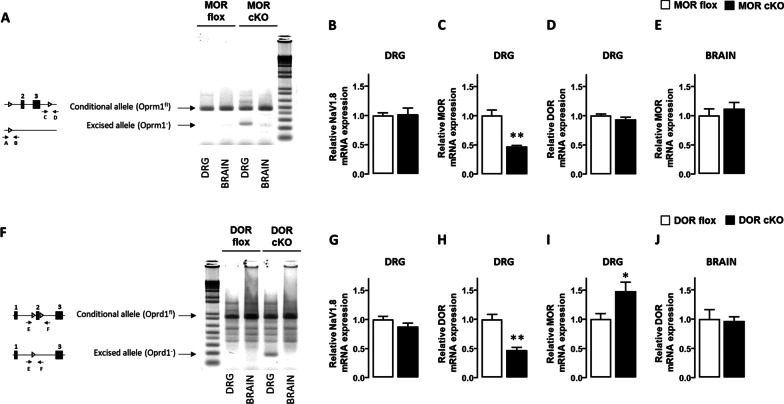
Analysis of the conditional knockout mice with specific deletion of either MOR or DOR in primary afferent Na_v_1.8-expressing neurons. **A** The scheme shows the *Oprm1*^fl^ conditional allele or “floxed” allele (*Oprm1*^fl/fl^) with exons 2 and 3 of the MOR gene flanked by two loxP sites and the excised allele (deletion of the exons 2 and 3) resulting from the expression of Cre recombinase under the control of the Na_v_1.8 sodium channel (*SCN10A*) gene promoter. Arrows indicate primers used to detect gene excision (A and B) and floxed allele (C and D) by PCR. PCR experiments show exon 2–3 deletion in DRGs but not in brain of MOR cKO mice. The two bands in DRGs result from gene excision in primary afferent nociceptive neurons (Na_v_1.8^+^ neurons) but not in other (Na_v_1.8-negative) cells. mRNA expression levels of Na_v_1.8 (*SCN*10A) (**B**), MOR (*Oprm1*) (**C)** and DOR (*Oprd1*) (**D)** were quantified by quantitative RT-PCR in DRGs from MOR cKO mice (black histogram) and their wild-type MOR flox littermates (white histogram). The absence of non-specific germline activation of the Cre recombinase was confirmed by quantifying MOR (*Oprm1*) mRNA level in brain samples (**E**). mRNA expression levels were normalized to wild-type MOR flox littermates (*n* = 5–6 mice/group). **F** The scheme shows the *Oprd1*^fl^ conditional allele or “floxed” allele (*Oprd1*^fl/fl^) with exon 2 of the DOR gene flanked by two loxP sites and the excised allele (exon 2 deletion) resulting from the expression of Cre recombinase under the control of the Na_v_1.8 (*SCN*10A) gene promoter. Arrows indicate primers (E and F) used to examine gene excision by PCR. PCR depicts exon 2 deletion in genomic DNA from DRGs but not brain of DOR cKO mice. The two bands in DRGs result from gene excision in primary afferent nociceptive neurons (Na_v_1.8^+^ neurons) but not in other (Na_v_1.8-negative) cells. mRNA expression levels of Na_v_1.8 (*SCN*10A) (**G**), DOR (*Oprd1*) (**H)** and MOR (*Oprm1*) (**I)** were quantified by quantitative RT-PCR in DRGs from DOR cKO mice (black histogram) and their wild-type DOR flox littermates (white histogram). The absence of non-specific germline activation of the Cre recombinase was confirmed by quantifying DOR (*Oprd1*) mRNA level in brain samples (**J**). mRNA expression levels were normalized to wild-type DOR flox littermates (*n* = 5–6 mice/group). Data are expressed as mean ± SEM. Statistical analysis was performed using Mann–Whitney *U* test. *p < 0.05, **p < 0.01 compared to wild-type littermate mice

To identify receptor(s) responsible for the opioid-induced analgesia at the later phase of the DSS-induced colitis, we used conditional knockout (cKO) mice in which the enkephalin opioid receptors mu (MOR) or delta (DOR) are specifically deleted in Na_v_1.8-expressing nociceptive neurons. cKO mice in which MOR (*Oprm1*) or DOR (*Oprd1*) genes are specifically deleted in nociceptors were generated by crossing *Oprm1*^fl/fl^ or *Oprd1*^fl/fl^ mice with mice that express Cre recombinase in Na_v_1.8-expressing neurons [16, 48]. As genetic background may impact pain assays, the cKO MOR or cKO DOR were compared to their respective Cre-negative MOR flox or DOR flox littermates with the same genetic background. As shown in Fig. 1, the cKO MOR or cKO DOR mouse lines are specifically deleted for MOR (Fig. 1A) or DOR (Fig. 1F) in peripheral nociceptive neurons but not in other tissue as brain, thereby ruling out a non-specific germline activation of the Cre recombinase. The absence of any alteration of Na_v_1.8 (*SCN10A*) mRNA expression levels in both cKO MOR (Fig. 1B) and cKO DOR (Fig. 1G) suggested that the targeted deletion of the opioid receptor genes did not impact Na_v_1.8 sodium channels in nociceptors. The two cKO mouse strains exhibited virtually similar 53–54% reduction of the targeted opioid receptors MOR (Fig. 1C) or DOR (Fig. 1H) in DRG neurons as assessed by quantitative PCR. The specific deletion of MOR in DRGs did not modify the expression of DOR (Fig. 1D), while the specific deletion of DOR was associated with a weak but significant up-regulation of MOR in DRGs (Fig. 1I). The similar expression levels of MOR (Fig. 1E) and DOR (Fig. 1J) mRNA in the brain of the cKO mice as compared to their floxed control littermates confirmed the absence of non-specific germline activation of the Cre recombinase in each cKO mouse strain.

**Fig. 2:**
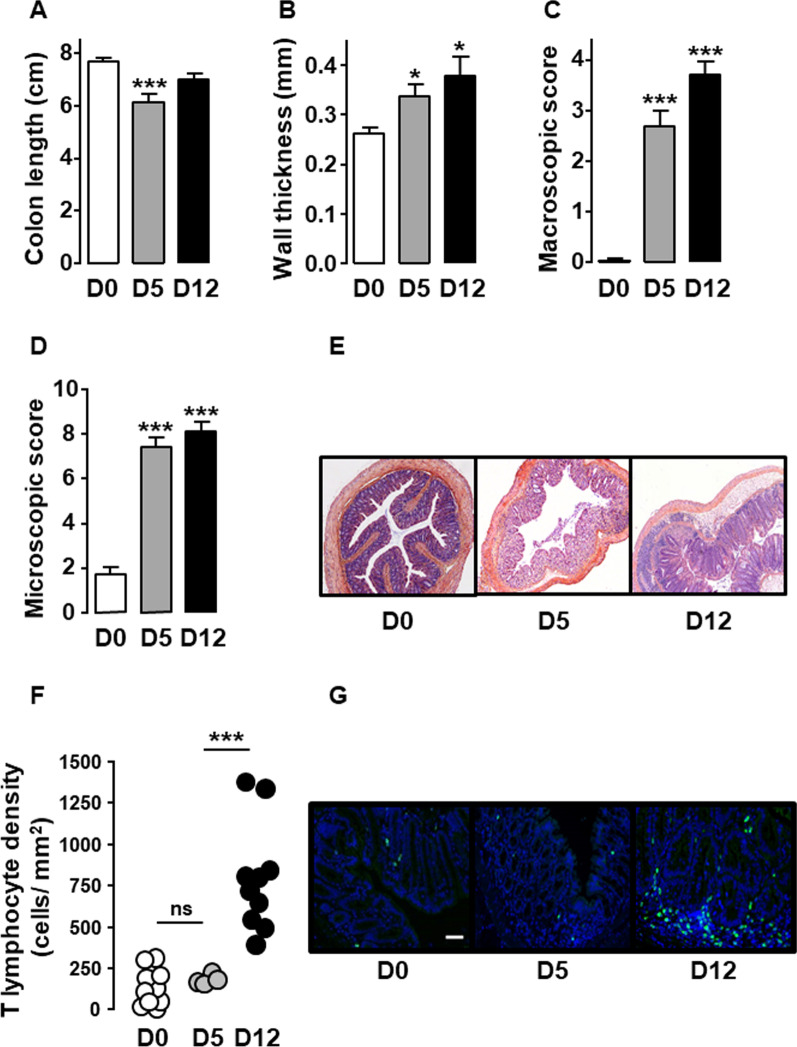
T lymphocytes accumulate within inflamed colonic mucosa upon DSS-induced colitis. Littermate wild-type mice untreated (D0, white), treated 5 days with 3% DSS (D5, grey) or treated with 3% DSS for 5 days and then with water until day 12 (D12, black) were examined for colitis severity including colonic length (**A**), wall thickness (**B**), macroscopic (**C**) and histological (**D**) colonic tissue damage (*n* = 10–20 mice/group). A representative histopathological analysis performed on H&E-stained colon sections in depicted in **E**. Untreated mice (D0) show normal epithelial architecture with neither cellular infiltration nor edema (**E**, left panel). Mice treated 5 days with 3% DSS (D5) show massive cellular infiltration, submucosal edema and epithelial architecture disruption (**E**, middle panel). Mice treated with 3% DSS for 5 days and then with water until day 12 (D12) show massive cellular infiltration, severe edemas (submucosal and subepithelial) and epithelial disruption (**E**, right panel). **F** Density of CD3^+^ T lymphocytes within *lamina propria* (each point, corresponding to one animal, represents the mean of four different histological examinations per slice) quantified per mm^2^ of tissue (*n* = 4 (D5) or 10 (D0 and D12) mice/group). A representative anti-CD3 immunostaining at day 0 (left panel), day 5 (middle panel) and day 12 (right panel) of the treatment is depicted in **G** (scale 50 µm). Data are expressed as mean ± SEM. Statistical analysis was performed using Kruskal–Wallis and subsequent Dunn’s multiple comparison tests. *p < 0.05, ***p < 0.001 compared to normal control mice (D0)

**Fig. 3:**
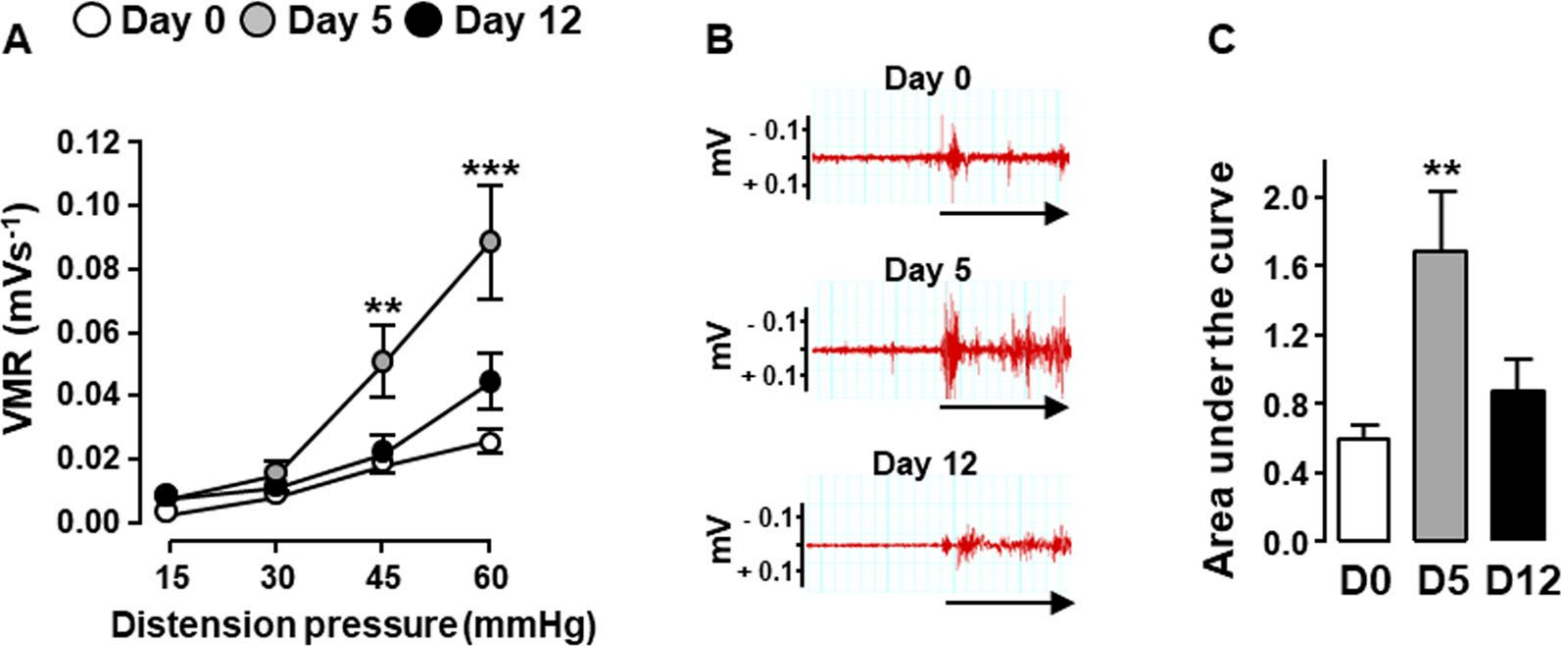
Visceral sensitivity normalizes on day 12 of the DSS-induced colitis. Visceral sensitivity was examined in littermate wild-type mice untreated (Day 0, white), treated 5 days with 3% DSS (Day 5, grey) or treated with 3% DSS for 5 days and then with water until day 12 (Day 12, black) by measuring visceromotor response (VMR) to colorectal distension (*n* = 9–18 mice/group) (**A**). **B** Representative traces of abdominal muscle contractions in response to 60 mm Hg colorectal distension in mice at day 0 (upper panel), day 5 (middle panel) and day 12 (lower panel) of the treatment. The arrows represent the 10 s of distension. Area under the curve (AUC) calculated by plotting individual VMR is shown in **C**. Data are expressed as mean ± SEM. Statistical analysis was performed using repeated-measures two-way ANOVA and subsequent Sidak’s multiple comparison tests (**A**) or Kruskal–Wallis and subsequent Dunn’s multiple comparison tests (**C**). **p < 0.01, ***p < 0.001 compared to normal control mice (D0)

We then confirmed that the MOR and DOR-floxed control littermates with a C57BL6/J-SV129Pas genetic background responded to DSS treatment as already shown in BALB/c mice [10]. As expected, after five consecutive days of treatment with 3% DSS, floxed mice developed colitis characterized by a significant reduction of colon length, an increase of wall thickness as well as macroscopic and microscopic tissue injuries (Fig. 2A–E) while T lymphocyte density within the mucosa remained stable as compared to untreated mice (Fig. 2F). This early phase of the disease was associated with visceral hypersensitivity (Fig. 3). As compared to mice treated 5 days with 3% DSS (Day 5), those receiving water for 7 additional days (Day 12) showed a significant increase in mucosal T lymphocyte density (Fig. 2F). Despite colitis-associated damage at day 12 of the DSS treatment, the increase in lymphocytes within the inflamed *lamina propria* was associated with a normalization of the visceral sensitivity as shown by the abdominal response to colorectal distension similar between DSS-treated and untreated mice (Fig. 3).

**Fig. 4:**
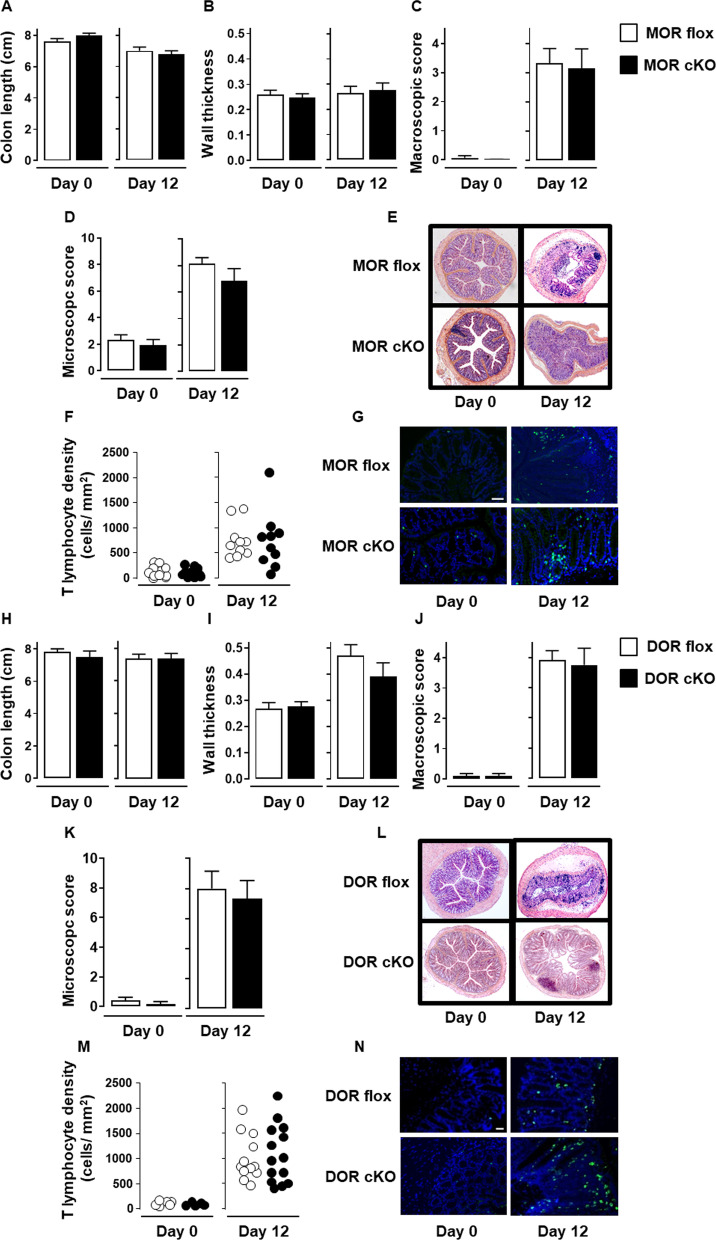
MOR or DOR deletion in Na_v_1.8-expressing sensory neurons does not alter neither colitis severity nor T cell infiltration within inflamed mucosa. Conditional knockout mice (black histogram) for either MOR (MOR cKO) (**A**–**G**) or DOR (DOR cKO) (**H**–**N**) were compared to their corresponding littermate wild-type floxed mice (MOR flox and DOR flox, respectively) (white histogram) in the basal conditions (Day 0) and on day 12 of DSS treatment for colitis severity (*n* = 7–8 mice/group) (**A**–**E** and **H**–**L**) and mucosal CD3^+^ T lymphocyte density (each point, corresponding to one animal, represents the mean of four different histological examinations per slice) (**F**, **G**; *n* = 10 mice/group and **M**, **N**; *n* = 6–14 mice/group). Representative histopathological H&E-stained colon sections (**E** and **L**) and anti-CD3 immunostaining (**G** and **N**) (scale 50 µm) at day 0 and day 12 of the DSS treatment for conditional MOR (**E**, **G**) and DOR (**L**, **N**) KO mice and their respective littermate wild-type MOR and DOR-floxed mice are shown. Data are expressed as mean ± SEM. Statistical analysis was performed using Mann–Whitney *U* test

**Fig. 5:**
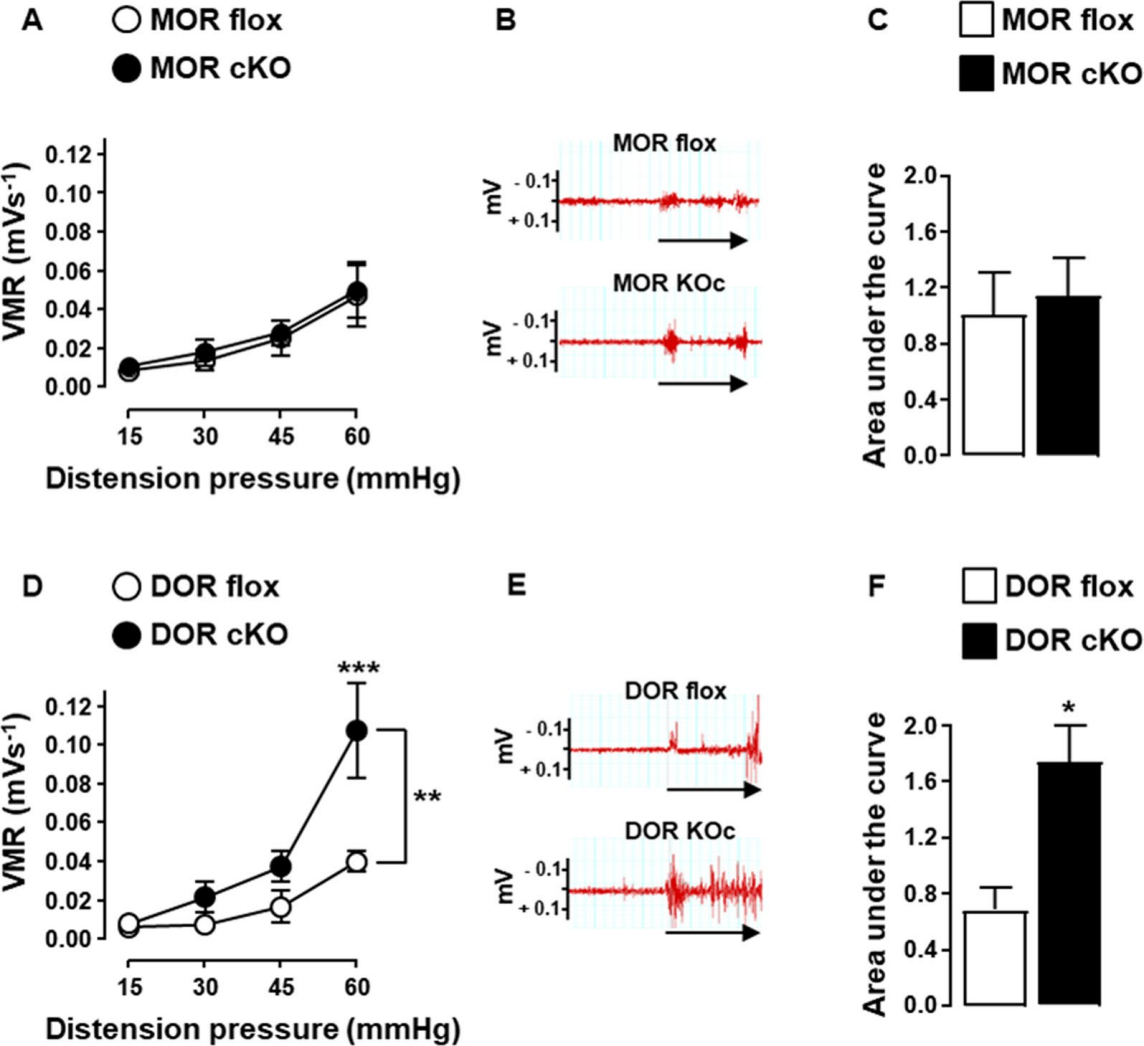
DOR deletion in Na_v_1.8-expressing sensory neurons abolishes analgesia on day 12 of the DSS-induced colitis. Visceral sensitivity of conditional knockout mice (black symbols) for either MOR (MOR cKO) (**A**–**C**) or DOR (DOR cKO) (**D**–**F**) was compared to their corresponding littermate wild-type floxed mice (MOR flox and DOR flox) (white symbols) on day 12 of DSS treatment (*n* = 5–7 mice/group). **A** and **D** Visceromotor response (VMR) to colorectal distension. **B** and **E** Representative traces of abdominal muscle contractions in response to 60 mm Hg colorectal distension in littermate floxed mice and conditional KO mice. The arrows represent the 10 s of distension. **C** and **F** Area under the curve (AUC) calculated by plotting individual VMR. Data are expressed as mean ± SEM. Statistical analysis was performed using repeated-measures two-way ANOVA and subsequent Sidak’s multiple comparison tests (**A** and **D**) or Mann–Whitney *U* tests (**C** and **F**). *p < 0.05, **p < 0.01, ***p < 0.001

**Fig. 6:**
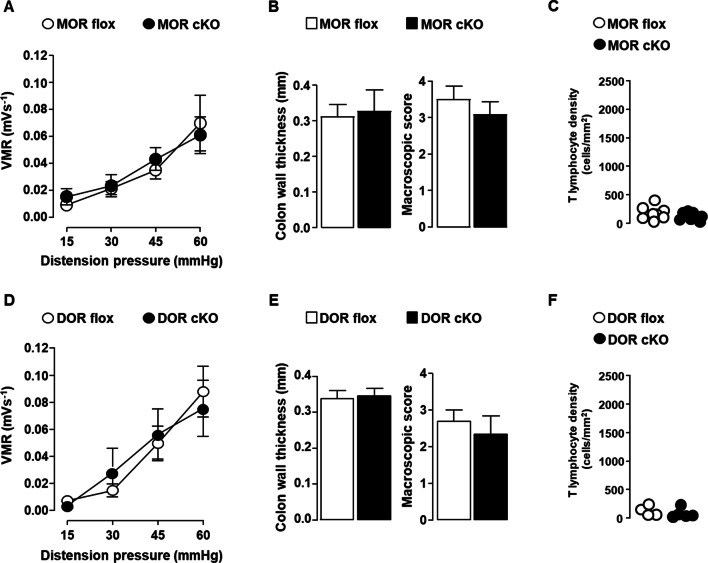
Deletion of MOR or DOR in Na_v_1.8-expressing sensory neurons does not impact inflammatory visceral pain in mice treated with 3% DSS for 5 days. Conditional knockout mice (black symbols) for either MOR (MOR cKO) (**A**–**C**) or DOR (DOR cKO) (**D**–**F**) were compared to their corresponding littermate wild-type floxed mice (MOR flox and DOR flox) (white symbols) (n = 7–10 mice/group) on day 5 of the 3% DSS treatment for visceral sensitivity (**A** and **D**), colitis severity assessed by colon wall thickness and macroscopic tissue damage (**B** and **E**) and mucosal CD3^+^ T lymphocyte density (each point, corresponding to one animal, represents the mean of four different histological examinations per slice) (**C** and **F**) (n = 4–8 mice/group). Data are expressed as mean ± SEM. Statistical analysis was performed using repeated-measures two-way ANOVA (**A** and **D**) or Mann–Whitney *U* test (**B**, **C**, **E** and **F**)

To identify which opioid receptor on nociceptors is involved in the spontaneous analgesic effect observed in the later phase of the disease, we compared conditional MOR or DOR knockout mice to their floxed control littermates on day 12 of the DSS-induced colitis. We first determined whether MOR and DOR conditional knockout mice differ or not from their floxed littermates for colitis parameters. As shown in Fig. 4, the deletion of MOR or DOR in Na_v_1.8^+^ sensory nerves did not alter neither the severity of colitis (Fig. 4A–E and H–L) nor the accumulation rate of T lymphocytes within the inflamed mucosa (Fig. 4F–G and M, N). The impact of the deletion of each opioid receptor on the sensitivity of mice to colorectal distension was then investigated at day 12 of the DSS-induced colitis. Whereas the loss of MOR in nociceptors had no effect on the visceral sensitivity, that of DOR resulted in the loss of analgesia (Fig. 5). To confirm that the activation of DOR was dependent on effector T lymphocytes infiltrating the inflamed mucosa, we then assessed DOR conditional knockout mice at the early phase of the disease. As depicted in Fig. 6, the deletion of DOR in Na_v_1.8^+^ sensory neurons, as that of the MOR, did not affect the visceral hypersensitivity observed at day 5 of the DSS treatment. This latter result was observed when T lymphocytes did not yet infiltrate the mucosa (Fig. 6C and F), in agreement with our previous study [36]. It supports the pivotal role of DOR expressed in Na_v_1.8^+^ nociceptors in the T lymphocytes-mediated endogenous control of inflammatory visceral pain.

## Discussion

T lymphocytes are key regulators of inflammatory visceral pain as exemplified in the dextran sulfate sodium (DSS)-induced colitis model [5, 6, 10, 46]. In this model, intestinal epithelium integrity disruption by DSS leads to the translocation into the mucosa of bacteria that first activate innate immune cells before T cell-mediated adaptive immune response takes place few days later. The two steps of the immune response match with the painful (acute) and painless (later) phases of the disease. In this colitis model, enkephalins locally released by T lymphocytes infiltrating the inflamed mucosa on the later phase of the disease normalize visceral sensitivity by acting on opioid receptors expressed in nociceptor endings [5, 7, 10].

Here, our study shows that peripheral DOR plays a major role in the endogenous regulation of inflammatory visceral pain in mice. Indeed, the targeted deletion of DOR in Na_v_1.8-expressing nociceptors prevents from the endogenous opioid-mediated analgesia at the latter phase of the DSS-induced colitis when T lymphocytes accumulate within the inflamed mucosa. Conditional knockout mice for MOR and DOR which are heterozygous for the Cre recombinase into the Na_v_1.8 locus preserve the expression of Na_v_1.8 channels in nociceptive neurons [42]. Accordingly, heterozygous Na_v_1.8-Cre recombinase mice display normal nociceptive inflammatory response [29]. Among the colonic afferents, MOR and DOR mRNA were found, respectively, in 38% and 46% of small-diameter neurons that have properties of nociceptors while 23% co-express MOR and DOR [18]. We identified DOR expressed in DRG neurons as the main targets in the endogenous regulation of colitis-induced visceral pain by using DOR conditional knockout mice that display a selective inactivation of *Oprd1* gene in small and medium size dorsal root ganglia (DRG) neurons while remaining active in the spinal cord and brain of the animals [16]. Our results obtained with a genetic approach are in line with a recent study showing that supernatants from colonic biopsies recovered from mice chronically treated with DSS inhibit nociceptor excitability via DOR but not MOR in vitro [22]. In these experiments, the inhibitory activity of colonic supernatants on the excitability of DRG neurons was prevented by antagonist for DOR but not MOR [22]. Of note, exogenous MOR agonist ligands were able to decrease neuronal excitability, showing that MOR is functional [22].

Both MOR and DOR are known to be locally upregulated in intestine under inflammatory conditions, a situation that could be responsible for the enhanced efficacy of agonist opioid ligands during colitis [31–33]. So, it has been shown that the MOR agonist DAMGO reduced excitability of nociceptive DRG neurons as assessed by patch clamp recording [44]. An observation recently substantiated, in the preclinical DSS-induced colitis model, by the peripheral analgesic effect of a novel MOR agonist preferentially active in the acidified environment of the inflamed colon [23]. Activation of DOR expressed on peripheral colonic nociceptors also induces potent analgesia in DSS-induced colitis model, endosomal signaling at DOR causing long-lasting inhibition of pain [22]. The beneficial effects of opioid agonists would also apply in the partial colon obstruction model as both analgesia and decrease in colon-specific DRG neuron excitability induced by Lactobacillus *reuteri* was reversed by the peripheral opioid receptor antagonist naloxone-methiodide [19].

Our observations indirectly corroborate the findings that mouse T lymphocytes mainly produce enkephalins, the most selective endogenous DOR ligands [3, 7, 11] and that colitis is made painful when the enkephalin-encoding *Penk* gene is deleted in T lymphocytes [5, 9].

Moreover, as previously shown for somatic inflammatory pain [12], our study indicates that even if both MOR and DOR are expressed and functional in sensory neurons innervating the colon [16, 18, 21–23, 31, 48], only DOR is relevant for the endogenous T cell-mediated analgesia in mice as a Na_v_1.8^+^ neuron-expressed opioid receptor.

The ongoing opioid crisis leads to propose innovations in the management of visceral pain. Thus, to avoid centrally mediated side effects and a better benefit/risk ratio, a number of strategies strictly targeting opioid receptors in periphery are under investigation [23, 37, 41]. MOR that is the main target of pharmacological drugs commonly used in intestinal inflammatory disorders is also the source of a number of serious side effects such as bowel dysfunction. Because DOR activation produces similar but milder side effects, the pharmacological targeting of DOR appears potentially more attractive [17]. In this context, the key role of DOR on primary afferents in the endogenous control of intestinal inflammatory pain opens new therapeutic opportunities for peripherally restricted DOR analgesics including, as already developed for MOR agonists, molecules acting at low pH [15, 41]. Of note, the local production of endogenous opioids by immune cells within the inflamed colon might also reduce tolerance to chronic treatment by preserving DOR signaling in peripheral sensory neurons [50].

## Conclusions

Taken together, our results suggest that the development of DOR agonists specifically active in periphery may improve effectiveness of opioid therapy in the treatment of chronic inflammatory visceral pain and dramatically reduce risk of developing addictive behavior.

## Data Availability

All data generated or analyzed during this study are included in this published article.

## Abbreviations

cKO: Conditional knockout
DRG: Dorsal root ganglia
MOR: μ-Opioid receptor
DOR: δ-Opioid receptor
DSS: Dextran sulfate sodium IBD: inflammatory bowel diseases
